# Phonological working memory and reading in students with dyslexia

**DOI:** 10.3389/fpsyg.2014.00746

**Published:** 2014-07-18

**Authors:** Carolina A. F. de Carvalho, Adriana de S. B. Kida, Simone A. Capellini, Clara R. B. de Avila

**Affiliations:** ^1^Speech Language and Hearing Department, Federal University of São PauloSão Paulo, Brazil; ^2^Speech Language and Hearing Department, State University São PauloMarília, Brazil

**Keywords:** working memory, dyslexia, language, reading, comprehension

## Abstract

**Purpose:** To investigate parameters related to fluency, reading comprehension and phonological processing (operational and short-term memory) and identify potential correlation between the variables in Dyslexia and in the absence of reading difficulties.

**Method:** One hundred and fifteen students from the third to eighth grade of elementary school were grouped into a Control Group (CG) and Group with Dyslexia (GDys). Reading of words, pseudowords and text (decoding); listening and reading comprehension; phonological short-term and working memory (repetition of pseudowords and Digit Span) were evaluated.

**Results:** The comparison of the groups showed significant differences in decoding, phonological short-term memory (repetition of pseudowords) and answers to text-connecting questions (TC) on reading comprehension, with the worst performances identified for GDys. In this group there were negative correlations between pseudowords repetition and TC answers and total score, both on listening comprehension. No correlations were found between operational and short-term memory (Digit Span) and parameters of fluency and reading comprehension in dyslexia. For the sample without complaint, there were positive correlations between some parameters of reading fluency and repetition of pseudowords and also between answering literal questions in listening comprehension and repetition of digits on the direct and reverse order. There was no correlation with the parameters of reading comprehension.

**Conclusion:** GDys and CG showed similar performance in listening comprehension and in understanding of explicit information and gap-filling inference on reading comprehension. Students of GDys showed worst performance in reading decoding, phonological short-term memory (pseudowords) and on inferences that depends on textual cohesion understanding in reading. There were negative correlations between pseudowords repetition and TC answers and total score, both in listening comprehension.

## Introduction

Many pieces of research confirm the importance of the integrity of the phonological system and the proper functioning of the processing of that information stored in a well-categorized manner, for the good performance in the decoding of written words (Ramus, 2001; Cain et al., 2004; Ramus and Szenkovits, 2008; Nevo and Breznitz, 2013). Therefore, components of phonological processing are fundamental to this initial process of decoding written words and will allow, in advance, automatic word recognition, enabling the direction of the skills for attention, memory and reasoning to understand the text (Wagner et al., 1987; Torgensen et al., 1994). Thus, the role of working memory on the comprehension of the written text is also known. Whatever the condition of decoding, the working memory allows the information to be kept until some issue with the sense of the text is resolved. Many other studies have investigated working memory capacity and its relations with the final product, which is reading comprehension (Daneman and Carpenter, 1980; Just and Carpenter, 1992; Seigneuric et al., 2000).

It seems logical to think that when the understanding ability is preserved, but reading is slow and inaccurate, being aware of the phonological working memory capacity is essential. In Dyslexia, these characteristics are present (Cain and Oakhill, 2006). Phonological processing deficits are recognized as the marker of Developmental Dyslexia. This deficit, associated with the difficulties of reading decoding, produces slowness and inaccuracy effects in word recognition (Snowling, 1981, 1995; Gathercole et al., 1999; Ramus and Szenkovits, 2008). And then, the slowness of reading should be compensated with good working memory capacities (and other cognitive and language skills) to reach the comprehension of the text, which is a common characteristic in dyslexics (Oakhill et al., 2003; Cain et al., 2004; Avila et al., 2009).

Working memory is often investigated by the performance on digit span test (WISC—III), whose phonological demand seems to be influenced by the predictability of stimuli. Since the phonological component is the greatest fragility in cases of Dyslexia, it would be interesting if the memory were assessed with low predictability linguistic stimuli, as are the pseudowords. This kind of stimulus could assess better the operational and retention conditions of the phonological information despite the risk of the slow processing of information, inherent to the disorder, affect the quality of the response.

This study is part of larger research on the ability of reading comprehension in Dyslexia. More broadly it was intended to understand how these deficits influence reading skills and for this, the operation of different abilities and skills that underlie and enable reading comprehension were studied.

In this research, the study of phonological short-term and working memory will be presented, along with their associations with reading comprehension, in dyslexia or in the absence of complaints of reading. But before that, decoding and listening comprehension was evaluated as a way of characterizing the two different groups of students who participated in the study.

## Purpose

To investigate parameters related to fluency, reading comprehension and phonological processing (operational and short-term memory) and identify potential correlation between the variables in Dyslexia and in the absence of reading difficulties.

## Material and methods

This study was approved by the Research Ethics Committee of *Universidade Federal de São Paulo* (UNIFESP/EPM) on April 3rd 2009—protocol n° 1731/08, and also by *Universidade Estadual de São Paulo—Faculdade de Medicina de Botucatu* (FM/UNESP/Botucatu-SP) on July 6th 2009—protocol n° 3277–2009.

### Sample

One hundred and fifteen students (67 of which girls), aged between 8 and 15 years old were selected. All participants were students enrolled between the third and eighth grade of Elementary education from different public schools.

The participants were grouped as follows:

- Group with Dyslexia (GDys): 17 students, who after speech-language pathologist, psycho-pedagogical, neuropsychological and neurological evaluation, were diagnosed with Development Dyslexia. Comorbidities such as attention deficit hyperactivity disorder were excluded.
- Comparison Group (CG): 98 children nominated by their teachers for good academic performance and no complaints or difficulties in learning how to read or write and aspects concerning school performance; no indication of having flunked, and having reached the expected standards for their schooling level in oral reading decoding tasks (Avila et al., 2009; Carvalho et al., 2009).
- In addition to criteria already defined for inclusion in both groups, others, including general aspects, have been considered to constitute the sample: absence of complaints or indicators of visual (uncorrected) and auditory sensory deficits, cognitive, behavioral and neurological disorders.

### Procedures

Students in the GDys were assessed at the Child Neurology Clinic—Learning Disorders, at FM/UNESP/Botucatu-SP. The students in CG were individually assessed in reserved classrooms at the school in class hours previously established by coordination and teaching staff.

In spite of the differences between location and condition of participants, the procedures, described below, were unique and used in identical ways for the assessment of both groups. The testing time for each procedure was determined, for both groups, according to the necessary time for conclusion of the proposed activities.

### Assessment of short-term and working memory

Among the processing of phonological components, the short-term and working memories were assessed by tasks of repetition of pseudowords (Cunha and Capellini, 2009) and repetition of digits in direct and reverse order (Wechsler, 1991). The pseudowords are linguistic items consisting of one to six syllables of extension based on Portuguese language patterns with no semantic correspondence. The list is made up of 24 items, The student's performance was analyzed by two different scores: (a) the received score corresponded to the number of syllables in the correctly repeated item, up to 50% of correct answers in the longest extension reached. For example, the student scored five when he or she repeated correctly up to 50% of the items presented with five syllables; (b) total number of correct answers.

For the test of Digit Span, the subtest from *Wechsler Intelligence Scale for Children—Third edition—*WISC-III (Wechsler, 1991) was applied, consisting of eight series of digits for repetition in direct order, and seven for reverse order. There is a gradual increase in the quantity of digits in each series. The student's performance was analyzed by three different scores: (a) the received score corresponded to the number of digits in the correctly repeated span, up to 50% of correct answers in the longest extension reached. For example, the student scored five when he or she correctly repeated up to 50% of the span items, with five digits presented for both attempts; (b) number of correct answers, separately, in direct and reverse order; (c) weighted score, corrected by a psychologist.

### Word and text decoding

In order to assess decoding, values of rate and accuracy parameters, observed in oral reading of words, pseudowords and texts, were considered. The specific tasks for oral reading were: reading items in isolation—words and pseudowords—and reading texts, used by Carvalho et al. (2009) and Kida et al. (2010). The lists of items were balanced in terms of extension, frequency and orthography. For the oral reading of texts, there was a text for each grade (Carvalho et al., 2009; Kida et al., 2010).

### Assessment of reading comprehension

Reading comprehension was assessed through the instrument proposed by Cain and Oakhill (1999), translated and adapted to Brazilian Portuguese. The instrument consists of two protocols: Protocol A, with four narrative passages, aimed at children from 8 to 9 years and 11 months old; and Protocol B, with three narrative passages, aimed at children 10 years or older. Each text presents six open questions which assess different types of cognitive processes and are grouped into: two questions of literal comprehension (LIT): understanding of explicit information in the text; two text-connecting (TC) inference questions: process through which the reader integrates the implicit information in order to establish cohesion between different phrases; two gap-filling (GAP) inference questions: process through which the reader uses his or her (previous) knowledge and the text content to fill information gaps and thus integrate the propositions of the text. The answers were scored 0, 1, or 2 points (incorrect, incomplete and complete answers respectively) according to standard answers established by the original instrument and by a board of three speech-language pathologists.

Each student was instructed to read the texts silently and attentively. They were also informed that right after reading, they should orally answer questions about what they had read. They were further informed that they would be allowed to reread the text if they wanted to before answering the questions.

### Assessment of oral comprehension

The relationship between oral and written language skills has been long accepted in studies of development. In order to read with adequate comprehension, one has to understand the language in its spoken form (Cain and Oakhill, 2008; Cain, 2010). Then, to better understand the reading comprehension, the listening comprehension was assessed using questions about a story that was told orally. All eight questions were formulated and grouped under LIT (4), TC (2), and GAP (2), similarly to the reading comprehension assessment. The students were instructed to listen to the story attentively so that they could understand it and answer, orally and immediately after the end of the narration, some open questions asked by the examiner. The answers were transcribed and scored 0, 1 or 2 points (incorrect, incomplete and complete answers respectively) according to standard answers established by a board of three speech-language pathologists.

### Data analyses

Mann-Whitney Test was conducted to compare the results obtained in assessments of decoding, oral and reading comprehension and working and short-term memory between students of CG and GDys paired by sex, age and school year, 1:1. Pearson Correlations were conducted, for each group separately, to measure the degree of linear dependence among the variables. Correlation values below 0.40 were considered low, between 0.40 and 0.80 were considered moderate and above 0.80 were considered high (Dancey and Reidy, 2006).

In the tests of text oral reading and of reading comprehension, different texts were used (appropriate for each grade) and, for this reason, Z scores were calculated for comparison among Groups. The 5% (0.05) significance level was adopted for application in the statistical tests.

## Results

Table 1 shows that in the comparison between student performance in tests of reading decoding, there was a statistically significant difference in the following investigated parameters: total reading time for words and pseudowords; text, pseudowords and word reading rate; text, pseudowords and words read correctly (percentage and score Z), and pseudoword and word accuracy. The performance of CG students was significantly better than that of GDys students in the three tests, which assessed aspects of reading decoding, except for the parameter total time of text reading, which did not show significant difference.

**Table 1:**
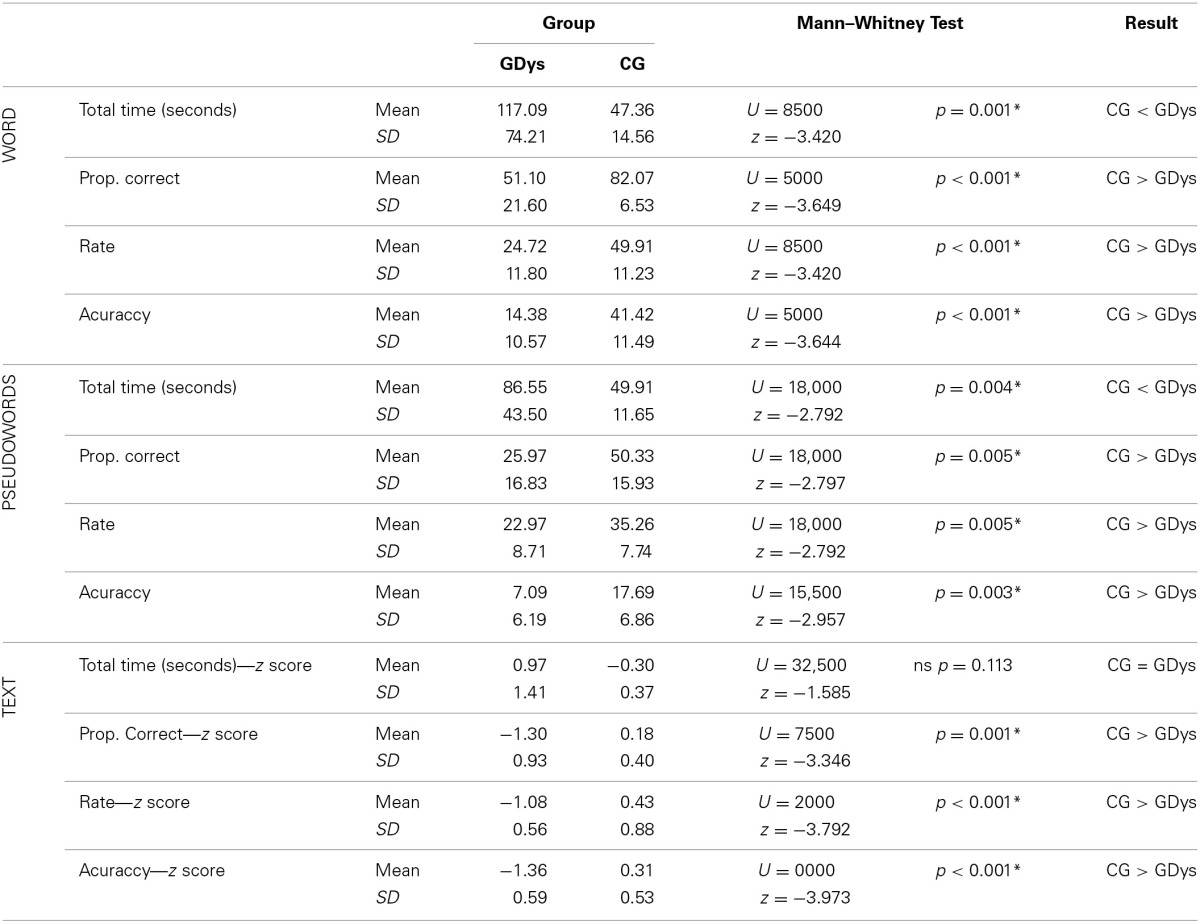
**Comparison of performances obtained in tests of reading decoding**.

SD, standard deviation; Prop. Correct, proportion of correct items. ^*^p < 0.05.

Observation of the analysis in Table 2 indicates that there was no statistically significant difference between CG and GDys in the answers to questions related to orally presented narration and when they answered literal and gap-filling reading comprehension questions. There was a difference in the comparison between the number of correct answers in the total score and the answers to questions, related to inferences necessary for connection of textual information, the best performance being from the CG.

**Table 2:**
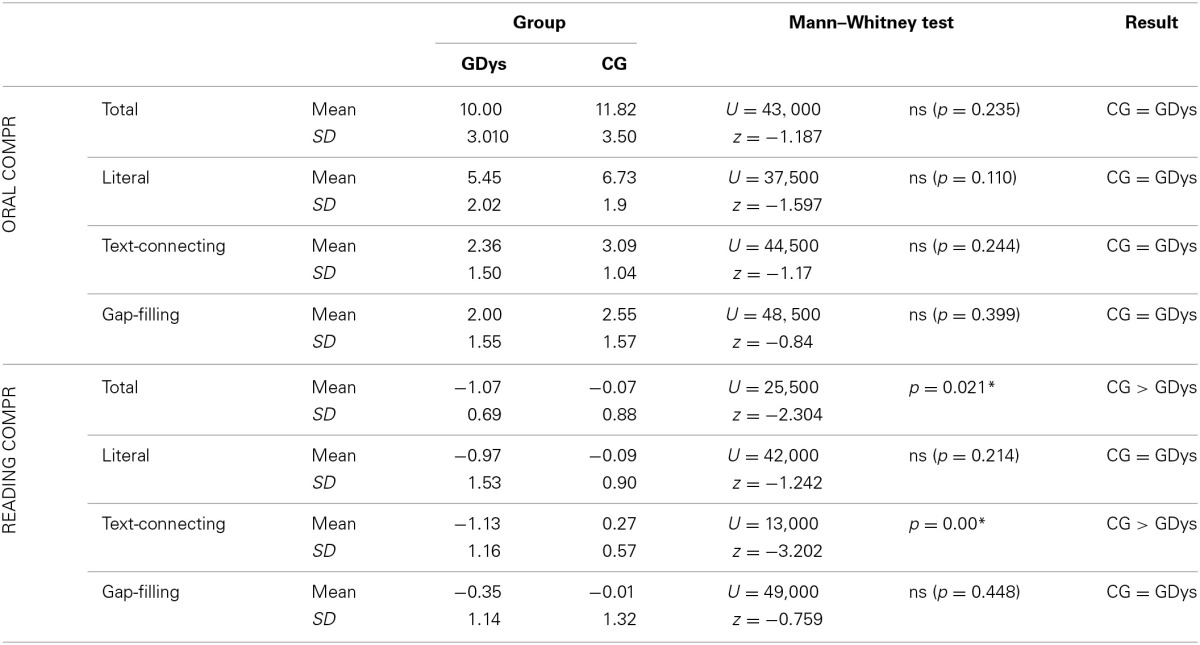
**Comparison of performances obtained in oral and reading comprehension tasks**.

SD, standard deviation. ^*^p < 0.05.

Table 3 shows that in the comparison between performances of the groups regarding the measurement of the extension in the pseudowords repetition test, the best result being from the CG. In the Digit Span Test, there was no difference between groups in respect of the total number of correct answers when repeating pseudowords and in any of the parameters.

**Table 3:** **Comparison of performances obtained in the test of Digit Span and Pseudoword Repetition**.

				**Grupo**		**Mann–Whitney Test**		**Result**
				**GDys**	**CG**			
Pseudoword repetition	Extension		Mean	5.09	5.91	*U* = 26,500	*p* = 0.009^*^	CG > GDys
			*SD*	0.94	0.30	*z* = −2.625		
	Correct answer		Mean	20.64	21.73	*U* = 44,500	ns (*p* = 0.279)	CG = GDys
			*SD*	2.20	1.01	*z* = −1.083		
Digit Span		Extension	Mean	4.45	4.73	*U* = 48,500	ns (*p* = 0.380)	CG = GDys
	DO		*SD*	0.69	0.78	*z* = −0.978		
		Total score	Mean	6.00	7.00	*U* = 41,000	ns (*p* = 0.185)	CG = GDys
			*SD*	1.10	1.73	*z* = −1.325		
		Extension	Mean	2.91	3.27	*U* = 41,500	ns (*p* = 0.110)	CG = GDys
	RO		*SD*	0.54	0.47	*z* = −1.598		
		Total score	Mean	3.64	4.09	*U* = 43,000	ns (*p* = 0.207)	CG = GDys
			*SD*	1.12	0.71	*z* = −1.261		
	DO + RO	Normed score	Mean	8.00	9.18	*U* = 45,500	ns (*p* = 0.315)	CG = GDys
			*SD*	2.45	2.27	*z* = −1.004		

*DO, direct order; RO, reverse order*.

* *p < 0.05*.

Tables 4, 5 presents the correlation values between the variables used in the study for the CG and GDys, respectively. For the CG, there were significant correlations between some of the oral reading parameters, answers to literal questions of oral comprehension, answers to text-connecting questions in reading; answers to gap-filling questions in reading with the performance of repeated pseudowords and digits. For GDys only significant correlation values of oral comprehension (total score and answers to literal questions of oral comprehension) and of reading comprehension (answers to gap-filling and to literal questions in reading) with working and short-memory parameters were found. Variables of oral comprehension (total score and answers to text-connecting questions of oral comprehension) were correlated with repetition of pseudowords, and variables of reading comprehension, with repetition of digits (total score and RO).

**Table 4:** **Correlations between variables—Comparison Group**.

		**Pseudoword repetion**		**Digit span**				
		**Extension**	**Total score**	**Extension DO**	**DO**	**Extension RO**	**RO**	**Total**
WORD READING	Total time	−0.224^*^	−0.190	0.011	0.002	−0.051	−0.101	0.158
	Prop. correct	0.182	0.262^**^	0.058	0.147	0.163	0.181	−0.027
	Rate	0.195	0.170	−0.006	−0.032	0.080	0.126	−0.155
	Accuracy	0.213^*^	0.215^*^	0.018	0.026	0.136	0.178	−0.124
PSEUDOWREADING	Total time	−0.205^*^	−0.126	−0.039	−0.021	−0.131	−0.144	0.084
	Prop. correct	0.131	0.255^*^	0.059	0.051	0.197	0.256^*^	0.000
	Rate	0.153	0.056	0.033	−0.009	0.136	0.139	−0.089
	Accuracy	0.160	0.180	0.064	0.034	0.218^*^	0.256^*^	−0.024
TEXT READING	Total time	−0.184	−0.183	−0.023	−0.043	−0.066	−0.069	−0.005
	Prop. correct	0.158	0.151	0.108	0.082	0.074	0.135	0.084
	Rate	0.248^*^	0.212^*^	−0.055	−0.028	0.100	0.094	0.020
	Accuracy	0.221^*^	0.205^*^	−0.042	−0.020	0.106	0.117	−0.002
READING COMPR	Total	−0.088	0.011	−0.046	0.010	0.148	0.167	0.038
	LIT	−0.115	0.076	−0.076	−0.027	0.117	0.155	0.041
	TC	−0.056	0.091	0.044	0.095	0.175	0.183	0.228^*^
	GF	−0.074	0.058	0.003	0.044	0.011	−0.034	0.395^**^
ORAL COMPR	Total	−0.027	0.057	0.133	0.167	0.197	0.156	0.042
	LIT	−0.013	0.026	0.200^*^	0.179	0.207^*^	0.170	0.049
	TC	−0.099	−0.057	0.003	0.035	0.191	0.149	0.026
	GF	0.074	0.154	0.024	0.101	0.068	0.039	−0.016

*DO, direct order; RO, reverse order; Prop. Correct, proportion of correct items; LIT, literal questions; TC, text-connecting questions; GF, gap-filling questions*.

* p < 0.05,

** *p < 0.001*.

**Table 5:** **Correlations between variables—Group with Dyslexia**.

		**PSEUDOWORD REPETION**		**DIGIT SPAN**				
		**Extension**	**Total score**	**Extension DO**	**DO**	**Extension RO**	**RO**	**Total**
WORD READING	Total time	−0.089	−0.342	0.423	0.357	0.099	0.218	0.179
	Prop. correct	0.132	0.288	−0.209	−0.303	−0.324	−0.414	−0.280
	Rate	0.023	0.254	−0.032	−0.125	−0.090	−0.188	−0.150
	Accuracy	0.055	0.256	0.017	−0.114	−0.184	−0.264	−0.184
PSEUDOWREADING	Total time	−0.038	−0.319	0.489^*^	0.369	0.124	0.203	0.119
	Prop. correct	0.067	0.379	−0.087	−0.102	−0.250	−0.254	−0.071
	Rate	−0.008	0.286	−0.202	−0.196	−0.174	−0.218	−0.113
	Accuracy	0.036	0.332	−0.018	−0.065	−0.237	−0.225	−0.096
TEXT READING	Total time	−0.144	−0.324	0.173	0.221	0.204	0.398	0.367
	Prop. correct	−0.039	0.264	−0.060	−0.184	−0.281	−0.309	−0.245
	Rate	0.151	0.084	−0.027	−0.263	−0.054	−0.140	−0.398
	Accuracy	0.061	0.348	−0.026	−0.114	−0.164	−0.275	−0.223
READING COMPR	Total	−0.058	0.081	0.158	−0.012	−0.208	−0.201	−0.201
	LIT	0.048	0.116	−0.213	−0.307	−0.459	−0.529^*^	−0.553^*^
	TC	0.212	0.160	0.065	−0.067	−0.242	−0.254	−0.303
	GF	−0.379	−0.121	0.452	0.375	0.327	0.435	0.565^*^
ORAL COMPR	Total	−0.437	−0.573^*^	0.185	−0.003	0.239	0.267	−0.277
	LIT	−0.280	−0.430	−0.084	−0.260	−0.082	−0.004	−0.337
	TC	−0.511^*^	−0.620^**^	0.477	0.208	0.413	0.431	−0.049
	GF	−0.204	−0.179	0.085	0.109	0.213	0.141	−0.147

*DO, direct order; RO, reverse order; Prop. Correct, proportion of correct items; LIT, literal questions; TC, text-connecting questions; GF, gap-filling questions*.

* p < 0.05,

** *p < 0.001*.

## Discussion

The comparison of performances in reading decoding showed the expected results and characterized the GDys with worse results compared to the CG in all activities related to decoding, except for the total time spent on reading texts. The similarity between the total times spent by both groups can be explained by the other comparison results in the assessment of reading comprehension, which showed that the GDys, at some level, understood the texts read. In other words, one can consider the influence of comprehension on parameters of reading fluency, as literature has shown (Oakhill et al., 2003; Cain et al., 2004; Avila et al., 2009). In general, even with low values of rate and reduced accuracy, the reading of the students from GDys was driven by the ability to integrate information, preserved in this group.

An important component of phonological processing, short-term and operational memory was assessed by repeating the sequence of pseudowords and digits In the repetition of pseudowords, GDys showed worse performance compared to CG as the extension of pseudowords increased and therefore, these results corroborate the literature (Snowling, 1981; Catts, 1986; Snowling et al., 1986; Kamhi et al., 1988; Wimmer, 1993; Stone and Brady, 1995). The deficit in the ability to retrieve items can be attributed to the interaction between the processes of short-term memory and phonological representation, stored in the long-term memory (Couture and Mccauley, 2000). According to the literature, in Dyslexia, the deficit in repeating pseudowords originates in poor accuracy of phonological representation in long-term memory (Elbro et al., 1994, 1998; Elbro and Jensen, 2005), or in inefficiency when accessing such representation (Wagner et al., 1987, 1994; Torgensen et al., 1994). Difficulties related to phonological representation and access to it impair discovery of phonemic elements in the pseudoword repetition task, since these must be segmented into their phonological units and analyzed on the basis of knowledge of phonologically similar units in long-term memory, and without having ever been previously articulated (Snowling, 1995; Nevo and Breznitz, 2013). Grivol and Hage (2011) noticed decrease in children's performance when repeating pseudowords as the number of syllables increased, and Couture and Mccauley (2000) obtained similar results in a study of children with phonological alterations.

Likewise, in this study the parameter that showed the difference between groups was the measurement of extension in the repetition test of pseudowords, showing better performance of students in the CG. However, no difference was observed between groups by measuring the total number of correct responses. Besides, the working memory, which in this research was evaluated by repeating digits in reverse order and which requires a different cognitive demand, has more predictable stimuli. Therefore, it is more closely related to storage and simultaneous processing of the information itself than to the quality of the phonological substrate; and perhaps that is why the difference between the groups' performances was not seen, as it was in the repetition of the digit span in direct order. Thus, it could be thought that the results found in this type of task, more closely related to the processing of reading comprehension (Cain et al., 2004), add weight to the hypothesis that, in Dyslexia, the integration mechanisms involved in text comprehension are preserved, in some way.

Reading inaccuracies observed in the GDys is related to inefficiency in processing phonological information (Snowling, 1981, 1995; Gathercole et al., 1999; Ramus and Szenkovits, 2008; Melby-Lervåg and Lervåg, 2012; Oliveira et al., 2012), which, in this study, was brought forward in the task of pseudoword repetition.

In relation to the performance in reading comprehension, differences were observed when comparing total score and answers to questions related to the inferences necessary to connect textual information with better performing of CG. No differences were observed between the performances of the groups to answer literal and gap -filling issues.

The comparative analysis of performance assessment that evaluated oral comprehension showed results that were consistent with the hypothesis that, in dyslexia, the greatest damage can be observed in the processing of phonological information.

The pattern of correlations established for each group was different. The analyses showed that the main difference found between these patterns could be observed in positive correlations between phonological memory and reading fluency parameters in CG, while in readers with dyslexia, these associations were not observed. This difference reinforces the evidence of the specific nature of phonological disorder in GDys.

## Conclusion

The analysis of the tasks that the groups were submitted to may show worse performance of the group with dyslexia in the parameters of reading fluency, as expected and phonological short-term memory in the repetition test of pseudowords, characterizing the phonological nature of the disorder. The absence of correlations between phonological memory and parameters of reading fluency in dyslexia support this feature.

### Conflict of interest statement

The authors declare that the research was conducted in the absence of any commercial or financial relationships that could be construed as a potential conflict of interest.
